# The Differential Effect of Metformin on Osteocytes, Osteoblasts, and Osteoclasts

**DOI:** 10.1007/s11914-023-00828-0

**Published:** 2023-10-05

**Authors:** Teun J. de Vries, Antonella S. Kleemann, Jianfeng Jin, Ton Schoenmaker

**Affiliations:** 1 Department of Periodontology, Academic Centre for Dentistry Amsterdam, University of Amsterdam and Vrije Universiteit, Gustav Mahlerlaan 3004, 1081 LA Amsterdam, The Netherlands; 2 Amsterdam University College, University of Amsterdam and Vrije Universiteit, Science Park 113, 1098 XG Amsterdam, The Netherlands; 3 Department of Oral Cell Biology, Academic Centre for Dentistry Amsterdam, University of Amsterdam and Vrije Universiteit, Gustav Mahlerlaan 3004, 1081 LA Amsterdam, The Netherlands

**Keywords:** Metformin, Diabetes, Osteoclasts, Osteocytes, Osteoblasts, AMPK

## Abstract

**Purpose of Review:**

Metformin is an anti-glycemic agent, which is widely prescribed to diabetes patients. Although its alleged role on bone strength has been reported for some time, this review focuses primarily on the recent mechanistical insights of metformin on osteocytes, osteoblasts, and osteoclasts.

**Recent Findings:**

Overall, metformin contributed to steering anabolic activity in osteocytes. It caused lower expression in osteocytes of the negative regulators of bone formation sclerostin and DKK1. Likewise, the osteoclastogenesis function of osteoblasts was also skewed towards lower RANKL and higher OPG expressions. Osteoblast lineage cells generally responded to metformin by activating bone formation parameters, such as alkaline phosphatase activity, higher expression of anabolic members of the Wnt pathway, transcription factor Runx2, bone matrix protein proteins, and subsequent mineralization. Metformin affected osteoclast formation and activity in a negative way, reducing the number of multinucleated cells in association with lower expression of typical osteoclast markers and with inhibited resorption. A common denominator studied in all three cell types is its beneficial effect on activating phosphorylated AMP kinase (AMPK) which is associated with the coordination of energy metabolism.

**Summary:**

Metformin differentially affects bone cells, shifting the balance to more bone formation. Although metformin is a drug prescribed for diabetic patients, the overall bone anabolic effects on osteocytes and osteoblasts and the anti-catabolic effect on osteoclast suggest that metformin could be seen as a promising drug in the bone field.

## Introduction

Though discovered just longer than 100 years ago in 1922, the biguanide metformin has been applied as an anti-glycemic agent since 1950 for type-2 diabetes. It has been used in the context of diabetes, where it helps to restore the body’s response to insulin by lowering the blood sugar produced by the liver and taken up by the intestines. Recent years have put extra claims on metformin such as an anti-aging and an anti-cancer drug, but also as a bone-protective drug [1]. Crucial to understanding metformin’s mode of action is likely its effects on mitochondria and on adenosine monophosphate activated kinase (AMPK) activity, as elaborated on in a recent review [1]. Evidence is presented that metformin may slightly improve bone quality of metformin users [2], but how it mechanistically affects cell activity remains obscure to some extent. A recent review has shown that the vast majority of studies on metformin and bone or on bone cells show a positive, anabolic effect [3••]: 19 out of 22 studies that have been investigated show a positive effect of metformin, whereas 2 had a no effect and one had a negative, anti-anabolic effect [3••]. Though not the scope of this review, we briefly mention the effects that metformin could have on other cellular processes and on a variety of diseases. In their review, Ala and Ala describe beneficial effects of metformin on biological processes such as senescence and inflammation [4]. At the disease level, metformin could have beneficial effects on bone diseases associated with excessive degradation such as osteoporosis and periodontitis but also on non-bone diseases such as inflammatory bowel disease and cardiovascular disease [4]. A recent study summarizes the beneficial effects in 15 studies using metformin in osteoarthritis in cell and animal studies [5]. Animal studies of osteoarthritis report reduced pain [5].

This review will primarily focus on the recently described mechanistical findings of how metformin interferes with the mode of action of the three major bone cells, i.e., osteocytes, osteoblasts, and osteoclasts, with the main emphasis on findings of the last 3 years. Thus far, focus has been on metformin’s action on osteoblasts. This review will further highlight the recent findings on osteocytes and osteoclasts. It attempts to elucidate how one molecule, with sometimes shared pathways that are affected, can have positive effects on osteocytes and osteoblasts, whereas osteoclasts are negatively affected by the same molecule. Importantly, two generic aspects of metformin should be mentioned. First of all, it is a polarized molecule, which means that it enters the cells through a membrane channel, in this case organic cation transporters solute carrier 22A (SLC22A is the gene; OCT 1 is the protein) [6–8]. Second, its major role is to lower glucose production in the liver, making tissues more sensitive to insulin [9]. Since most of the in vivo mouse model systems and cell culture are devoid of insulin, some of the mechanisms of the effects of metformin on bone cells described below may elucidate additional modes of action of metformin or could be artificial to some extent, since some of the studies may use artificially high and not clinically relevant doses [9].

## Metformin Activates Osteocytes to Induce Bone Formation

Osteocytes are the orchestrators of bone metabolism, since their signals lead to either activation of osteoblasts where more bone is needed or activation of osteoclasts when bone resorption is required, such as in case of bedrest, space flight, or other disused bone [10]. Since the osteocyte, with its more than 100 cellular extensions per cell is embedded in bone, and by definition is a non-dividing cell, cell models are rare. Mouse derived MLO-Y4 osteocyte-like cell line is frequently used, since this cell line represents various osteocyte characteristics [11]. For the remains, one can study osteocytes’ response in dissected bones [12] or in tissue sections of mouse models.

The role of metformin on osteocyte biology has been examined in three recent studies [14–16]. One of the studies analyzed whether the adverse effects of the amino acid homocysteine on bone density could be reversed [14]. In humans, high serum concentrations of homocysteine are associated with bone loss [13]. It was shown that in the context of high levels of homocysteine, the addition of metformin reduces osteocyte apoptosis [14]. The widely used osteocyte cell line MLO-Y4 was used to demonstrate this effect. Metformin’s protective action was likely mediated through adenosine monophosphate activated kinase (AMPK), since an AMPK inhibitor canceled the protective effect of metformin [14]. AMPK functions as an energy sensor that coordinates multiple protective and energy-conserving signaling pathways, including the pathways activated by caloric restriction [1]. In a model using rats that were fed on a high fructose diet as a model for diabetes, it was shown that metformin prevents loss of trabecular osteocytes [15]. In probably the most extensive study on metformin and osteocytes, using a poly-ethylene osteolysis model, it was shown that metformin protects bone by reducing the expressions of the negative bone formation regulators sclerostin (SOST) and Dickkopf-related protein 1 (DKK-1) that are typically secreted by osteocytes [16], thereby providing the biological clue for bone formation. Another study focused on the osteoclastogenesis markers that are expressed by osteocytes. The biological clues for bone degradation were towards less bone resorption, since osteoprotegerin (OPG) expression was increased whereas receptor activator of nuclear factor kappa ligand (RANKL) expression was decreased by metformin [17••]. Also in this study, it was demonstrated that the beneficial effects of metformin could be attributed to AMPK activation, since inhibition of AMPK nullified the positive effects of metformin. In this inflammatory osteolysis model, it was further shown that metformin reduces the levels of inflammatory cytokines interleukin-6 (IL-6) and tumor necrosis factor-α [17••]. Together, these three studies indicate that metformin has a protective and anabolic role on osteocytes, lowering apoptosis, lowering osteoclastogenesis mediators, lowering inflammation mediators, and contributing to anabolic processes [14–16].

## Metformin and Osteoblasts: Metformin Induces Osteogenic Differentiation

The osteoblast is, like the osteocyte, also a mesenchyme-derived cell type. It is the cell type that produces bone and can be activated by its family member the osteocyte. In vitro studies of osteoblasts use human mesenchymal stem cells or related cells such as periodontal ligament fibroblasts, mouse calvarial osteoblasts or cell lines such as human cancer cell lines MG63 or U2OS or mouse embryonal cells such as MC3T3-E1 [20, 21].

With regard to metformin, the osteoblast has been studied most extensively, and an excellent recent review has described its anabolic effects [3••]. At the cell level using two osteoblast-like cancer cell lines U2OS and MG63, metformin was shown to stimulate alkaline phosphatase expression and mineralization [18••]. Also transcription factor Runt-related transcription factor 2 (Runx2) was upregulated, as well as bone matrix proteins collagen I, bone sialoprotein and osteocalcin [18••]. It further caused increased phosphorylation of mitogen-activated protein (MAP) kinase p38, altering the state of this protein to the form that is known to interact with Runx2, which is important for transcription of osteogenic genes. In a mobility assay, it was shown that both osteoblast-like cell lines migrated faster in the presence of metformin [18••], indicating that metformin is also involved in migration. A recent study identified various osteoblast lineages in a diabetes mouse model [19]. It was shown that type-2 diabetes suppresses the energy metabolism of osteoblasts, along with their differentiation. Addition of metformin to the diet of these mice ameliorated bone loss, with an increase in all bone formation markers, together with a restoration of osteoblastic function in vivo [19]. Likewise, they mimicked the glycolysis enhancement of metformin by overexpressing the glycolysis mediator hypoxia induced factor 1alpha (Hif1α) specifically in osteoblasts [19]. Also in these experiments, the bone formation parameters alkaline phosphatase, Runx2, and Collagen-1 were restored, as was confirmed in experiments with osteoblast-specific overexpression of another glycolysis mediator 6-Phosphofructo-2-Kinase/Fructose-2,6-Biphosphatase 3 (Pfkfb3) [19]. In a model where apoptosis was induced in MC3T3‑E1 osteoblasts with H_2_O_2_, which damages mitochondrial membranes, it was shown that metformin protected against apoptosis by upregulating sirtuin 3 (SIRT3), which is a stabilizer and protector of DNA damage [20]. In another study with the MC3T3-E1 osteoblast cell line, cells were exposed to high glucose in combination with metformin. Here, metformin increased proliferation of these cells, caused more alizarin red staining as an indication of mineralization, concomitant with higher expression of Runx2 and Osteonectin. Also, Wnt-1 and β-catenin were upregulated, showing that metformin positively affects the Wnt-signaling pathway [21]. Also Ma et al. [22] could correlate metformin to increase in Wnt-signaling. They studied a key regulator of Wnt-signaling, lowering glycogen synthase kinase-3β (GSK3β), which is a mediator of the degradation of β-catenin. Using bone marrow-derived mesenchymal stem cells as source for osteoblast precursors cells, they were able to show that the beneficial effects of metformin on mineralization and on the mRNA expression of Runx-2 and bone matrix proteins Collagen I and Osteonectin were lowered when GSK3β was silenced using siRNA transfection. As proof of Wnt signaling, phosphorylated β-catenin was lower in cells that were transfected with GSK3β antisense RNA [22]. Finally, to show interactions between AMPK and GSK3β, it was shown that lowering AMPK phosphorylation with a specific inhibitor lowered GSK3β phosphorylation [22]. In a study using human umbilical cord-derived mesenchymal stem cells as precursor cells of osteoblasts, it was also shown that these human-derived primary cells display enhanced osteogenesis under the control of metformin [23]. In their experiments, metformin neither affect apoptosis nor proliferation. However, osteogenesis was increased, concomitant with increased expression of Runx2, alkaline phosphatase and osteonectin, which was demonstrated both at the mRNA and protein level. Metformin induced the phosphorylation of mammalian target of rapamycin (mTOR) and growth factor AKT, which was reduced when adding an inhibitor of the P13Kinase, a protein that is upstream of the cell’s central energy and physiology pathway of mTOR [23]. As described above for osteocytes [17••], osteoblasts are also susceptible to the regulation of genes that regulate osteoclastogenesis. Mai et al. [24] described that metformin increases OPG expression and downregulates RANKL both in mouse calvarial osteoblasts and in MC3T3-E1 cells. Inhibitors of AMPK nullified metformin’s effect, suggesting that metformin’s mode of action is through AMPK [24]. Finally, they showed in ovariectomized rats that metformin upregulated OPG and downregulated RANKL, resulting in lower numbers of osteoclasts in the metformin-treated ovariectomized rats [24].

Apart from these cell biological mechanistic approaches, metformin was also incorporated in a carrier that filled large bone defects [25]. Metformin proved to be beneficial in bone healing. The bone grafts showed improved cell migration and differentiation compared to scaffolds without metformin.

Taken together, all studies on the effect of metformin on osteoblasts and their mesenchymal precursors show that metformin is beneficial for bone formation.

## Metformin Reduces Osteoclast Formation and Activity

The multinucleated bone degraders, osteoclasts, differentiate from mononuclear precursors of the monocyte/macrophage lineage. Within bone, osteocytes play an important role in their differentiation by the expression of RANKL [26]. In vitro models commonly make use of mouse bone marrow [27] or human blood monocytes in the presence of macrophage colony stimulating factor (M-CSF) and RANKL, or use osteoblast lineage cells in co-culture with blood monocytes [28••]. Mouse macrophage cell line RAW 264.7 is also frequently used as a cell line to study osteoclast formation [18••].

Metformin’s effect on the formation and activity of osteoclasts has only recently been documented, both in animal studies and in vitro [18••, 28••, 29–31]. A recent study analyzed the effect of metformin on both osteogenesis and osteoclastogenesis using tooth-derived periodontal ligament fibroblasts and an array of osteoclast formation assays [28••]. Periodontal ligament fibroblasts display osteoblast-like features such as high alkaline phosphatase expression and are indispensable for both osteogenesis and osteoclast formation. In contrast to any effect on osteogenesis, Tao and colleagues [28••] found that metformin inhibits osteoclast formation and activity in all assays. Regarding a possible mechanism, it was shown that metformin diminished the expression of RANKL and M-CSF by periodontal ligament cells as important osteoclastogenesis genes in co-culture assays with peripheral blood mononuclear cells [28••]. In line with the lower osteoclast counts, osteoclast genes tartrate-resistant acid phosphatase (TRACP) and fusion gene dendritic cell-specific transmembrane protein (DC-STAMP) were also downregulated [28••]. In monocyte cultures that were driven to osteoclast formation with M-CSF and RANKL, metformin downregulated RANK, the receptor of RANKL. Metformin also inhibited the formation and activity of osteoclasts in an assay with monocytes that were cultured with M-CSF and RANKL, both on cortical bone slices and on plastic [28••]. Similar anti-osteoclast effects were reported by Park et al. [18••]. Using RAW 264.7 cells as osteoclast precursors, metformin dose-dependently inhibited the formation of multinucleated cells, in line with a lower expression of the osteoclast markers non-receptor tyrosine kinase c-Src, TRACP, and Cathepsin K at the protein level [18••]. In a rat model for ischemic osteonecrosis, metformin was shown to protect against ischemic osteonecrosis by lowering osteoclast activity and maintaining osteocyte and osteoblast activity [18••]. Using mouse bone marrow cells as precursors, inhibitory effects of metformin on osteoclast formation and gene expression were also described by Chen et al. [29]. Metformin dose-dependently inhibited osteoclast formation, along with downregulation of osteoclast genes osteoclast-associated receptor (OSCAR), Cathepsin K and TRACP. It was shown that metformin decreased the phosphorylated extracellular signal kinase (ERK), indicating that metformin’s action was MAP kinase dependent. These findings were confirmed by Guo et al. [30], who also demonstrated in mouse bone marrow cells that metformin caused a decrease in osteoclast formation. This was established by cell counts of multinucleated cells and by a decreased expression of osteoclast markers TRACP, Calcitonin Receptor (CTR), Cathepsin K, and DC-STAMP. The addition of metformin maintained a high level of p-AMPK, by either reducing the ATP:AMP ratio by inhibiting the electron transport chain or by directly phosphorylating it [31]. RANKL alone caused a decrease in p-AMPK. However, metformin did decrease the phosphorylation of ERK and nuclear factor of activated T-cells NFAT-c1, both important signaling molecules for osteoclast differentiation. In an osteoarthritis mouse model, metformin prevented bone loss [30].

Another recent study researched the effect of metformin on bone and osteoclast parameters both in osteoporotic and diabetic postmenopausal women and in ovariectomized mice [32]. Serum markers for osteoclast activity TRACP and carboxy-terminal cross-linked telopeptide of type 1 collagen CTX-1 were lower in women receiving metformin. Furthermore, metformin showed to have a beneficial effect on bone parameters such as the T-scores of assigned vertebrae that were measured [32]. As in the female postmenopausal patients, OVX-treated mice receiving metformin had lower serum TRACP and CTX-1 values as well, and bone parameters were better in the metformin receiving group [32]. The osteoclast formation capacity of osteoclast precursors in vitro from mice that had received metformin was compromised, indicating that metformin not only affects naive osteoclast precursors that were not previously exposed to metformin, but also has an inhibitory effect on the osteoclast precursors that were exposed to it in vivo [32]. Mechanistically, Xie et al. [32] could link the decreased osteoclast formation and activity to decreased autophagy present in the precursors. Various autophagy markers were downregulated by metformin. Together, these studies on metformin and osteoclasts, using a variety of models and species, uniformly show that it inhibits the formation and activity of osteoclasts.

## Concluding Remarks

**Fig. 1:**
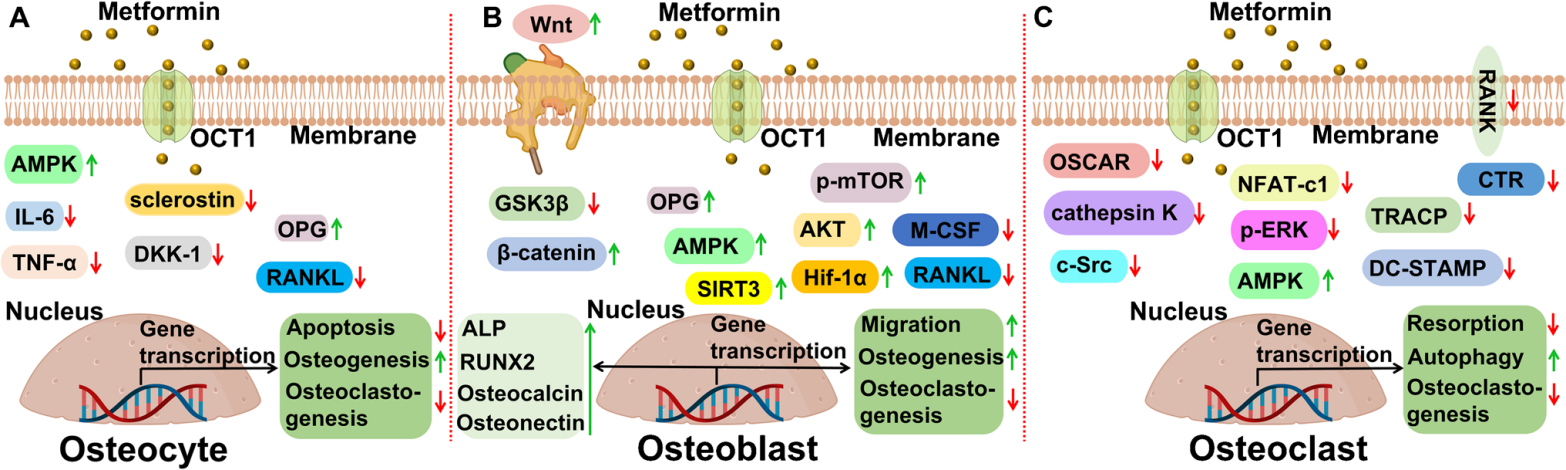
Effect of metformin on osteocytes, osteoblasts, and osteoclasts. Summary of the findings described in this paper. **A** Effect of metformin on osteocytes. **B** Effect of metformin osteoblasts. **C** Effect of metformin on osteoclasts. Ligth green boxes: cell processes that were influenced by metformin; other boxes: genes that were affected, either stimulatory (green arrow up) or inhibitory (red arrow down)

The effects of metformin on bone cells are intriguing. The studies summarized show anabolic effects on osteocytes and osteoblasts, whereas osteoclast formation and activity are inhibited, both in vitro and in in vivo models (see Fig. 1 for a summary on metformin’s effect on all three cell types, emphasizing cell processes). A common denominator is the beneficial effect of metformin on AMPK phosphorylation in all three cells. AMPK, as modulator of the energy level of a cell, could therefore influence the energy threshold required for proper cell differentiation. In case of the mesenchymal osteocytes and osteoblasts, it could result in the appropriate level required for osteogenic differentiation. In case of the monocyte-derived osteoclasts, it could lead to too low levels of energy, resulting in exhausted cells that require more energy for the proper functioning or differentiation. Concerning the effect of metformin on differentiation parameters, it is probably still a black box. It still remains unclear *how* it interferes with the initiation of differentiation pathways as seen in osteoblasts and the shutting down of differentiation seen in the osteoclast lineage. The elucidation of precise fine-tuning of metformin mechanisms, with emphasis on how it binds and to which regulators and how this influences the fate of bone cells, is the challenge for future research. In the future, metformin could be considered as an orphan drug, meaning a drug that responds to a general public health need. This study has clearly shown that bone cells are affected differentially, resulting in bone formation and suppression of bone degradation. Whether such a skewed balance is beneficial for bone strength, given the required coupling of osteoclasts and osteoblasts, remains to be seen. The effect of metformin on osteocytes is probably the key for future research, since metformin’s action is both pro-osteogenic (less sclerostin, less, DKK-1) and anti-osteoclastogenic (more OPG, less RANKL, less inflammatory markers IL-6 and TNF-α, Fig. 1).

## Data Availability

Not applicable, it is a review. All literature can be consulted to assess whether it was correctly reported on.
